# Quantifying Grass Coverage Trends to Identify the Hot Plots of Grassland Degradation in the Tibetan Plateau during 2000–2019

**DOI:** 10.3390/ijerph18020416

**Published:** 2021-01-07

**Authors:** Yaqun Liu, Changhe Lu

**Affiliations:** 1 Key Laboratory of Land Surface Pattern and Simulation, Institute of Geographic Sciences and Natural Resources Research, Chinese Academy of Sciences, Beijing 100101, China; yaqun_liu@163.com; 2 College of Resources and Environment, University of Chinese Academy of Sciences, Beijing 100049, China

**Keywords:** grassland change, Mann-Kendall test, Sen’s slope, climate variability, grassland protection policy, grazing intensity

## Abstract

Grassland covers 54% of the Tibetan Plateau (TP) and suffered overgrazing and degradation problems during past decades. To alleviate these problems, a series of policy measures have been implemented during recent two decades and inevitably caused changes of the grassland. To this end, this study quantitatively analyzed the grassland changes and the effects of reduced grazing intensity, and identified the hot plots of grassland degradation in the TP during 2000–2019. The grassland status was indicated by the Fractional Vegetation Cover in the green grass period (GP), i.e., FVC_GP_, and its changes and spatial variations were detected by analyzing the FVC_GP_ trends and their distribution, using the Mann–Kendal, Sen’s Slope, and ArcGIS buffering methods, and data of the MOD13Q1 Collection 6 products and other sources. The results showed that 62.12% of the grasslands were significantly increased in the FVC_GP_, and 28.34% had no apparent changes. The remaining 9.54% of the grassland significantly decreased in the FVC_GP_, mainly occurring in the areas nearby roads, rivers, and lakes, and distributed mostly in a point pattern. Of the total FVC_GP_ decreased grassland area, 27.03% was clustered and identified as the hot plots of grassland degradation in six main regions. Decreased grazing intensity and increased precipitation contributed to the increase of grassland FVC in the TP, while local overgrazing could be the main cause of the FVC decrease. To strength the grassland restoration in the TP, the government supports and supervision should be enhanced to further mitigate the grassland pressure of animal grazing, particularly in the hot plot areas of degradation.

## 1. Introduction

Grassland covers about 40% of the global land surface [1], provides vital ecosystem services [1,2], and plays an essential role in food security, biodiversity maintenance, and sustainable development [3,4]. However, during recent decades, half of the global grasslands were degraded due to anthropogenic and natural factors [2,3,5]. As one of the major rangeland regions in the world, the Tibetan Plateau (TP) was also experiencing serious grassland degradation [6,7,8,9], resulted in declines of productivity, biodiversity, soil carbon and nutrients, and related socio-economic functions [9,10,11,12]. Grassland degradation has become the main threat to the ecological security of the TP [8,13,14].

The grassland degradation in the TP occurred mostly during 1980s to the early of 21st century, as mainly caused by anthropogenic factors, particularly overgrazing, and shows high spatial heterogeneity [8,14,15,16,17]. Some studies reported that 50.9% of the grassland in Naqu of North Tibet was degraded in 2001 [6], and 36.1% of the grassland in the water source area of the Three Rivers (Yellow, Yangtze, and Lantsang Rivers) was degraded in 2004 [7]. To alleviate the grassland degradation, the Chinese government has implemented a series of policy measures to reduce the grazing pressure of the TP since the early 21st century [18,19,20]. These include financial supports to the herdsmen to reduce the grazing intensity and to adopt fenced grazing, as to achieve the forage–livestock balance. Several large projects have been implemented, such as the program of returning pasturage to natural grassland in 2003 [8], the protection plan of the Three Rivers nature reserve in 2005 [14], and the protection and construction plan of Tibetan ecological security shelter in 2009 [20]. These programs have been implemented for more than ten years; therefore, an assessment should be necessary to understand the grassland dynamics and to identify where the grassland is still under degrading, contributing to the improvement of grassland protection and restoration policies to achieve the sustainable management in the TP.

Grassland degradation involves multiple aspects, such as grassland coverage and productivity declines, soil deterioration, and losses of dominant species [2,21,22,23]. Due to data limitations, the coverage decline is often used as an indicator to identify grassland degradation, since it is much more sensitive to grazing intensity and can be easily detected by remote sensing (RS) data [24,25,26]. Many studies found that overgrazing causes grassland coverage reduction [8,21,24], but moderate grazing can increase grassland coverage, productivity, and biodiversity [19,26]. Therefore, detecting grassland coverage trends and the spatial variations can reveal the grassland dynamics and help to identify the areas where the grassland is restoring or degrading, as induced particularly by changes in the grazing intensity during a short to midterm period. Remote sensing imagery data have the real-time, objective and accurate ground information, and are consistently recorded [8,21,27], and thus can be based to derive vegetation coverage, i.e., the Fractional Vegetation Cover (FVC), and then to identify its change trends for certain period [21,25,28]. Of the various RS based indices including the Enhanced Vegetation Index (EVI) and Soil Adjusted Vegetation Index (SAVI), the Normalized Difference Vegetation Index (NDVI) is mostly used to estimate FVC [28,29]. Several studies indicate that the NDVI time series data of GIMMS-NDVI3g (8 km resolution, 1981–2015), SPOT-VEG (1 km, 1998–2014), and MODIS (250–5600 m, 2000–present) can be used to evaluate the vegetation growth status and productivity [30,31,32], and to find the evidences of grassland degradation over the past decades [8,14,33]. So, this study took the change trends of FVC in the main growing period to indicate the grassland dynamics during the study period of the protection policies implemented, and in particular, to identify the hot plots of grassland degradation where the grassland FVC was significantly decreasing and spatially clustered, as to service the determination of key areas that should be deserved more efforts to control the grassland degradation.

Recently, several studies analyzed the recent changes of grassland FVC in the TP, but the results differed largely in the change amplitude, trend, and pattern [8,34,35]. Some studies indicate that most (61.73–89.96%) grassland in the TP increased in the FVC, and a small proportion at 9.04%, 38.27%, and 16.00% was decreasing, based on the results from the SPOT-VEG data during 2000–2012 [34], MODIS Collection 5 (C5) data during 2000–2015 [8] and MODIS C6 product data during 2000–2018 [35], respectively. However, two other studies got much different results, i.e., in only 48.37% and 36.71% of the grassland, the FVC increased while in 51.63% and 43.29% decreased, as obtained from GIMMS-NDVI3g imagery data during 2000–2012 [34] and 2000–2015 [8], respectively. These inconsistences could be mainly caused by the difference in the data resolution and time span, and also the sensor shifts or degradation [36,37,38,39,40]. Use of low resolution imagery data may cause inaccurate interpretation of grassland change trends, particularly in mountain areas [38,41]. For GIMMS-NDVI3g and SOPT-VEG series data, the sensors were not consistent, which shifted from AVHRR 7 to AVHRR 9, 11, 14, 16, and 17, and shifted from SOPT-4 to SOPT-5 [31,32,38]. Although the MODIS sensors have not been changed, the C5 products have introduced a systematic downward trend due to sensor decline [8,39,40]. In general, MODIS C6 is a more reliable product for identifying grassland changes and degrading trends compared to GIMMS-NDVI3g, SPOT-VEG, and MODIS C5 products [8,39], because its sensors have not shifted and the influences of sensor degradation and cloud pollution have been eliminated [39,40].

The specific aims of this study are to detect the spatial variation of grassland dynamics during the recent period of 2000–2019, and to identify the hot plots of grassland degradation in the TP, taking the annual average of FVC in the green grass period (GP), i.e., the main growth reason, as the indicator, and the up-to-date MOD13Q1 C6 (250 m resolution) products as the main data source. In addition, the causes of grassland changes and the contribution of the policy measures to the grassland restoration were analyzed. Finally, policy implications for sustainable grassland management were discussed.

## 2. Materials

### 2.1. Study Area

The Tibetan Plateau (TP) is known as the third pole and is the water source area of several big rivers, including the Yangtze River, the Yellow River, the Ganges-Brahmaputra River, the Lantsang-Mekong River, and the Nu Jiang-Salween River; therefore, it is called as “the water tower of Asia”. It is dominated by alpine grassland ecosystem, and provides not only livestock products for local people, but also habitats for rare wild animals and essential services of water and soil conservation. As the ecosystem is sensitive and vulnerable to climate changes and human activities, the TP has been listed as one important priority region of China’s ecological security shelters [14,42], to protect the natural environment and to mitigate the intervention of human activities.

**Figure 1:**
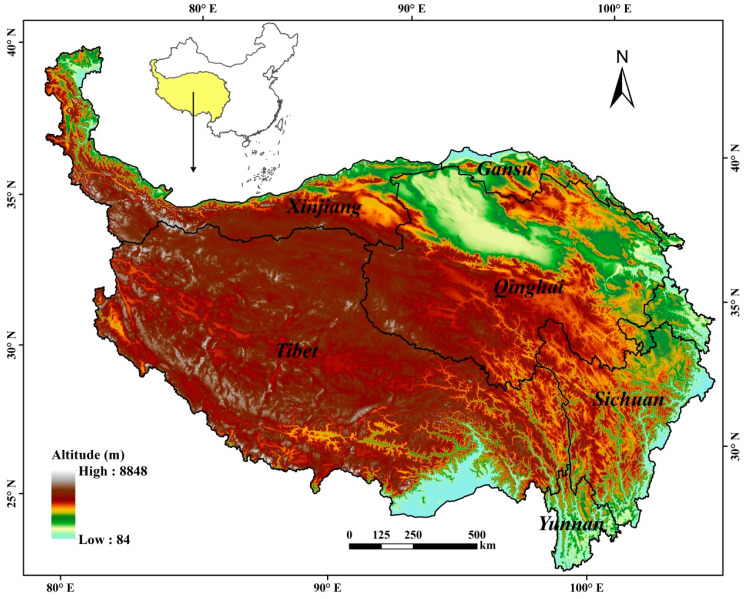
Location and altitude of the Tibetan Plateau.

The TP is located in southwestern China and covers an area of 268.32 Mha. It is the world′s highest geographical unit, and the major part is above 4000 m a.s.l. (Figure 1) and dominated by semi-arid alpine climate. From southeast to northwest, with the altitude increasing, the annual average temperature decreases from above 15 °C to below 0 °C, and the annual average precipitation drops from above 1000 mm to below 50 mm [13]. During the past 50 years, the TP showed a much faster (twice the world average) warming trend [42], and became more humid [8,16]. Influenced by elevation and climate, the ecosystems show an apparent spatial variation. In the southern and eastern mountain areas of the TP margin zones, the altitude is mostly below 3000 m a.s.l., and the ecosystem types are subtropical forests, broad-leaf and coniferous forests, crops, and grass vegetation. In the main part of TP, the ecosystems are dominated by sub-alpine and alpine grass vegetation and used for animal grazing.

### 2.2. Data Sources

Multiple sources data were collected, including NDVI data, land use data, DEM and slope data, climate data, and livestock data for the whole TP. The NDVI data for 2000–2019 was obtained from the MODIS MOD13Q1 C6 (NDVI-C6) products [43], used to calculate the FVC. The data have a spatial resolution of 250 m and a temporal resolution of 16 days.

The land use data in 2018 were obtained from the Resources and Environment Data Cloud Platform of Chinese Academy of Sciences [44]. It was compiled by visual interpretation of the 30 m resolution Landsat images, and the overall accuracy exceeds 90% [45].

The DEM and slope data at the spatial resolution of 30 m were collected from the Aster Global Digital Elevation Model (ASTER GDEM) version 2 dataset [46]. The vector data of roads, rivers and lakes were downloaded from the 91 satellite map platform [47] and used to calculate the distances to roads and to water areas.

The climate data (precipitation and temperature) for 2000–2017 at 93 stations in the grassland areas were collected from China Meteorological Data Service Center [48].

The livestock data for 2000–2018 were obtained from China statistical yearbooks [49]. Total animal number was converted to sheep-unit (SU), in which one sheep or goat equals one SU, and one large animal of yak, cow and horse equals 4.5 SUs according to the Agricultural Industry Standards of China (NY/T635-2015) [50]. In order to check the grassland changes and the effects of grassland protection policies, field observations on grassland growth status by reconnaissance surveys, and interviews of 66 herdsmen in northern TP were done in July 2019 and August 2020.

## 3. Methods

Grass coverage is an effective indicator of grassland growth status [28,29], and sensitive to changes of grazing intensity [19,24]. In this study, the grass coverage was defined as the annual average of FVC in the main growth season in which the grass is green, named as the green grass period (GP). The methodological procedure includes following steps: the first is to map the grassland distribution and to calculate its FVC. From the land use map of 2018, we extracted the grasslands (including shrub grassland) in the TP and calculated their FVC for all 16-day’s NDVI images during 2000–2019, and then obtained the grassland distribution by excluding the part of which the mean maximum FVC during 2000–2019 was below 5%. The second is to estimate the mean annual FVC in the GP (FVC_GP_), and the third is to detect the trends and change amplitude of FVC_GP_. The fourth is to identify the hot plots of grassland degradation, and the fifth is the causal analysis of grassland changes.

### 3.1. Calculation of FVC

The *FVC* was calculated with the dimidiate pixel model, a simple and effective model for the linear decomposition of mixed pixels [28,29,51]. It assumes that the NDVI value of one pixel is the area-weighted sum of the NDVI for vegetation-covered and no vegetation parts (i.e., bare land). The formula is as follow.

(1) $$FVC=\frac{NDVI_{pixel}-NDVI_{soil}}{NDVI_{veg}-NDVI_{soil}}$$

where *NDVI_pixel_* is the pixel NDVI value. *NDVI_soil_*, and *NDVI_veg_* refer to the NDVI value of no vegetation and fully vegetated pixel, as calculated based on the grassland NDVI values for the cumulative proportion of 5% and 95%, respectively.

The annual FVC_GP_ was obtained by averaging the FVC of all 16-day’s images in the GP year by year. The GP was estimated using the mean 16-day’s FVC during 2000–2019 (to reduce the interference of cloud pollution and climate fluctuations), by defining it as the continuous period in which the 16-day’s FVCs all exceed 1/3 of the annual maximum. For a specific pixel, same duration and beginning-ending dates of the GP were used, and for different regions, varying lengths of the GP were used to match the real grass growing season, as the GP varies greatly over spatial in the TP due to the influence of elevation and climate conditions. The ArcGIS (Environmental Systems Research Institute, Redlands, CA, USA) [52] and R project software (R Foundation, Vienna, Austria) [53] were used to identify the GP duration and to calculate the mean annual FVC_GP_ for each pixel. As previous studies were normally based on a constant period (e.g., May–September) for whole region to calculate the FVC in the TP [8,14,34], for comparisons, we also selected May–September as the period to calculate the FVC, named as FVC_MS_.

### 3.2. Detection of FVC_GP_ Trend

The trend and change amplitude of FVC_GP_ and FVC_MS_ for each pixel during 2000–2019 were calculated using the Mann-Kendall (M-K) test [54,55] and the Sen’s slope [56,57] non-parametric methods, which are widely used for the trend analysis in vegetation, climate, hydrology, agriculture, and other fields [58,59,60,61,62], based on the “trend” package in the R project software [53]. The M-K formulae are shown in Equations (2)–(5), and the Sen’s slope in Equations (6) and (7).

(2) $$Z=\left\{ \begin{array}{l} \frac{S-1}{\sqrt{Var\left(S\right)}},\;S>0\\ 0,\;S=0\\ \frac{S+1}{\sqrt{Var\left(S\right)}},\;S<0 \end{array}\right.$$

(3) $$S=\sum_{i=1}^{n-1}\sum_{j=i+1}^{n}sgn\left(X_{j}-X_{i}\right)$$

(4) $$sgn\left(X_{j}-X_{i}\right)=\left\{ \begin{array}{l} 1,\;X_{j}-X_{i}>0\\ 0,\;X_{j}-X_{i}=0\\ -1,\;X_{j}-X_{i}<0 \end{array}\right.$$

(5) $$Var\left(S\right)=\frac{n\left(n-1\right)\left(2n+5\right)-\sum_{i=1}^{m}t_{i}\left(t_{i}-1\right)\left(2t_{i}+5\right)}{18}$$

where *n* is the number of years in the time series, and *m* is the number of bound data groups. *X_i_* and *X_j_* are the *i*-th and *j*-th data values, respectively. *t_i_* is the length of the *i*-th bound data group, and *sgn* is the signum function. *Z* is the statistical value of the M-K test. For a confidence level *p*, *Z* value should satisfy the formula of |Z| > Z_1-p/2_. The positive or negative *Z* value indicates an increasing or a decreasing trend, respectively.

(6) $$FS=Median\left(\frac{FVC\left(j\right)-FVC\left(i\right)}{j-i}\right)$$

(7) $$FS\%=FS\times \frac{n}{\sum_{i=1}^{n}FVC\left(i\right)}\times 100$$

where *FS* and *FS%* refer to the annual change amplitude and rate of *FVC*, respectively. *FVC (i)* and *FVC (j)* refer to the *FVC* values for year *i* and *j (0 <= i < j <= n)*, respectively. *n* refers to the number of years in the study period, and *Median* is the median function.

### 3.3. Classification of Grassland Changes

Taking the change trend of annual FVC_GP_ as the indicator of grassland dynamics during 2000–2019, we classified the grasslands into 3 categories, named as increased (IN), not changed (NC) and decreased (DE), based on the Z and FS% values (Table 1). According to the M-K test, when the |Z| value exceeds 1.96 (the confidence is above 95%), the trend is significant, when it is between 0.675 and 1.96 (confidence is 50–95%), the trend is insignificant, and when it is below 0.675 (confidence is lower than 50%), no trend exists [59,60,62]. Based on these rules, the grasslands with the FVC_GP_ in significantly increasing/decreasing trends were deemed as IN/DE category, and those without showing change trends in the FVC_GP_ were classified as NC. The grasslands with the FVC_GP_ in insignificant trends were classified as IN, DE, and NC for the FS% > 0.25%, <−0.25%, and −0.25% < FS% < 0.25%, respectively. In this classification, when the annual change amplitude is below 0.25%, the trend was considered insignificant [63,64,65].

### 3.4. Identification of Grassland Degradation Hot Plots

In this study, the hot plots of grassland degradation were specified to the areas where the DE grassland was highly clustered, i.e., the areas concentrated by the grassland showing a significantly decreasing trend in the FVC_GP_. It did not include the grasslands that were under degrading of editable grasses but its FVC_GP_ was increasing during the study period due to non-editable weeds invasion [19,26]. The hot plots were identified using the clustering method of the Anselin Local Moran’s I indicator (Moran′s I), commonly used for analyzing spatial clustering characteristics of crop production, disasters, disease transmission, urban crime, etc. [66,67]. The formulae are as below:

(8) $$I_{i}=\frac{\left(n-1\right)\left(x_{i}-\bar{x}\right)}{\sum_{j=1,j\neq i}^{n}{\left(x_{j}-\bar{x}\right)}^{2}}\times \sum_{j=1,j\neq i}^{n}w_{ij}\left(x_{j}-\bar{x}\right)$$

(9) $$Z_{i}=\frac{I_{i}-E\left[I_{i}\right]}{\sqrt{V\left[I_{i}\right]}}$$

where *I_i_* and *x_i_* refer to the Moran’s I statistic and value for pixel (or grid) *i*, respectively. $\bar{x}$ is the mean of neighboring pixels, and *w_ij_* is the spatial weight between pixel *i* and *j*. *n* is the total number of the pixels in a statistical unit. *Z_i_* is the statistical significance of the *I_i_*. *E[I_i_]* and *V[I_i_]* refer to the average and variance of the *I* values for all pixels in a statistical unit, respectively. The positive or negative *I* value indicates that a statistical unit has clustering or dispersion pattern. The hot plots are the statistical units with statistically significant positive Moran′s I values. Thus, based on the *I* and *Z* values, the clustering patterns of grassland degradation were divided into two classes, i.e., hot plots (*I* > 0 and *Z* > 1.96) and non-hot plots (*I* ≤ 0 or *Z* ≤ 1.96).

### 3.5. Spatial Analyses of Grassland Change Trends

Grassland accessibility affects the grazing intensity, and thus causes differences of grassland changes and degradation over spatial, as evidenced by previous studies [8,14]. Therefore, detecting the spatial variation of grassland trends can help understanding the change or degrading causes. In this study, the buffering and geostatistical analysis tools in ArcGIS [52] were used to calculate the spatial distribution of FVC_GP_ trends, to find the variation with distance to roads and water areas (lake and rivers), and variation with slope steepness and dry–wet conditions.

### 3.6. Analyses of Climate and Grazing Intensity on Grassland Changes

Climate (precipitation and temperature) and grazing intensity are recognized as the main factors influencing the grassland changes in the TP [8,16,24,26]. In this study, their relevance to the grassland FVC_GP_ changes was quantified using the Pearson’s correlation analysis [3,8]. To reduce the interferences from other factors, the influence of precipitation and temperature were carried out at station scale. A window area comprising 3 × 3 pixels surrounding each meteorological station was selected and its annual mean FVC_GP_ was obtained for the correlation analysis. The analysis was done by taking the annual FVC_GP_ change (FC), i.e., the value in (*i* + 1)-th year minus that in *i*-th year, as dependence variable, and the annual change of precipitation (PC), and temperature (TC) as independent variables, using the data at the 93 window areas surrounding the stations during 2000–2017. For the influence of grazing intensity, the correlation analysis was done at provincial level for Tibet and Qinghai, and at county level for representative counties (i.e., Menyuan, Zhouqu, Rutog, Dege, Qumarlêb, Chindu, Basu, Kangding, Seni, and Damxung) using the annual average data of FVC_GP_ and grazing intensity during 2000–2018.

## 4. Results

### 4.1. Spatial Distribution of Grassland and Its Green Grass Period

**Figure 2:**
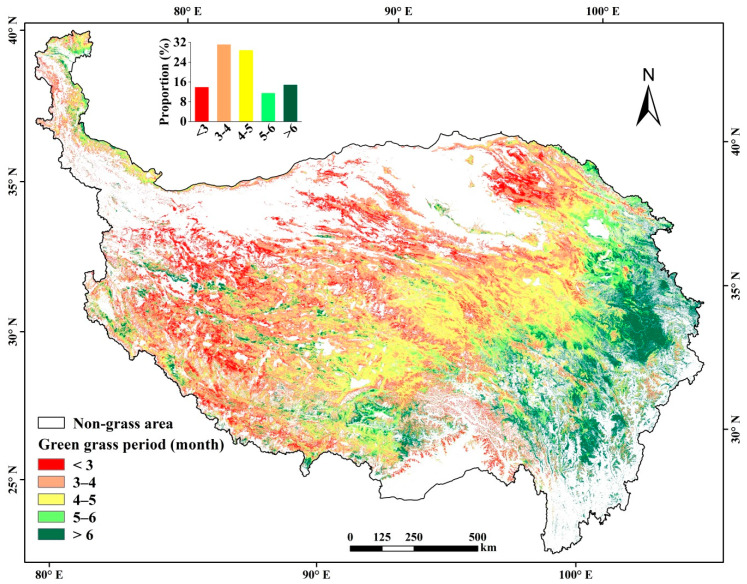
Spatial distribution of average green grass period (GP) in the TP during 2000–2019.

The grassland was totaled 145.22 Mha, covering 54.07% of the TP, mostly distributed in the high plateau and mountain areas above 3000 m a.s.l. (Figure 2). Jointly influenced by the low temperature and limited precipitation, the GP of grasslands was mostly (85.20%) less than six months (Table 2) and showed an increasing trend from northwest to southeast (Figure 2). In the northwestern TP where the annual temperature is normally below 0 °C and annual precipitation below 200 mm, the GP was mostly less than four months, and mean FVC_GP_ was lower than 25%. Moving to southeastwards, the climate is becoming warmer and wetter, and thus the GP apparently increased to over six months, and the mean FVC_GP_ increased to over 50% in eastern and southern TP. In northwestern plateau, some small areas in a zonal distribution are covered by wetland meadow and shrub-grass vegetation, and showed a longer GP than six months.

### 4.2. Spatial Variation of FVC_GP_ and FVC_MS_ Trends

**Figure 3:**
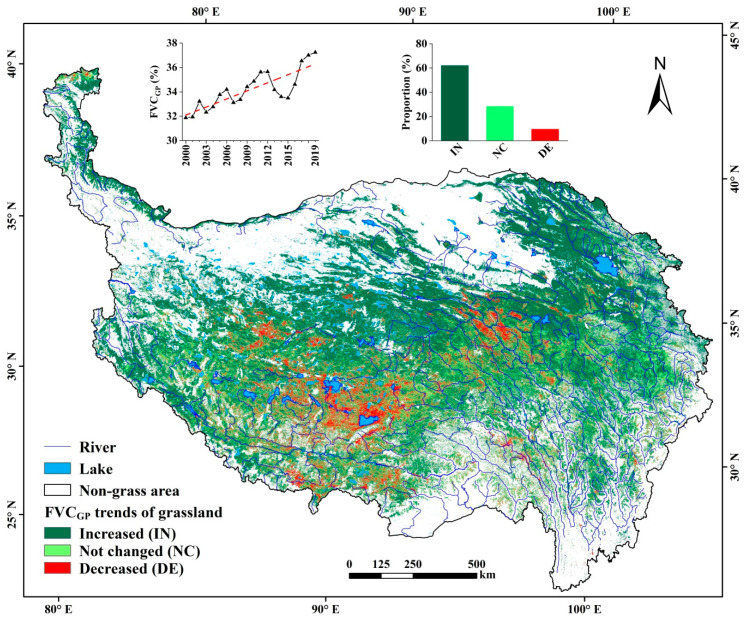
Spatial distribution of trends in the FVC_GP_ of grasslands during 2000–2019.

During 2000–2019, the mean annual FVC_GP_ of grasslands for the entire TP showed an increasing trend, from 31.87% in 2000 to 37.24% in 2019, increased by 0.65%/year (Figure 3). Specifically, 62.12% of the grasslands (IN) were obviously increased in the FVC_GP_, which were largely distributed in the natural reserve areas including the Qilian Mountains of northeast TP and the northern Qiangtang Plateau (Figure 3). The mean FVC_GP_ of this IN grassland type was the lowest (Table 3), but its increase trend was mostly obvious, at the rate of 1.24%/year (R^2^ = 0.8425) from 2000 to 2019. In 28.34% of the grasslands (NC), the FVC_GP_ fluctuated annually in a narrow range, without showing an evident trend. In the remaining 9.54% of the grasslands (DE), the mean FVC_GP_ decreased at the rate of −0.83%/year (R^2^ = 0.7434), which were mainly distributed in the river valleys and basins in south Tibet and west Qinghai plateaus (Figure 3).

**Figure 4:**
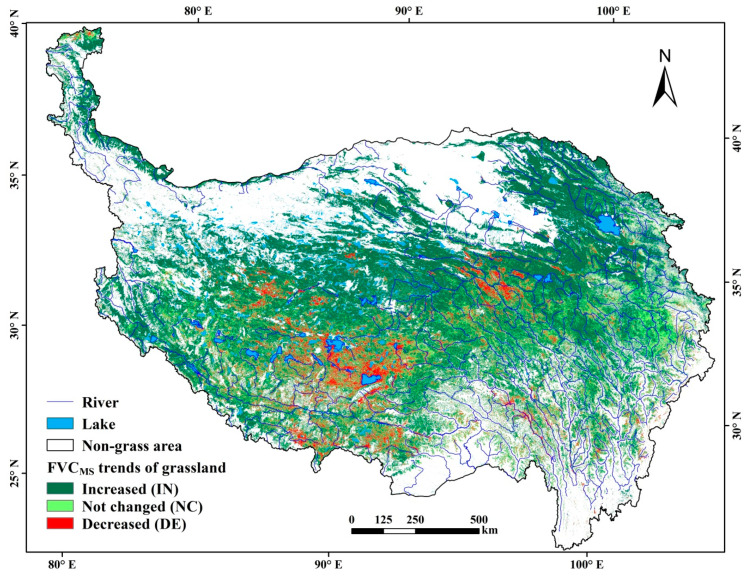
Spatial distribution of trends in the FVC_MS_ (May–September) of grasslands during 2000–2019.

The FVC_MS_ trends showed some differences from the FVC_GP_ (Figure 4). By spatial analyses, it was found that 83.36% of the trends were consistent (Table 4). For the grasslands with decreased FVC, the area covered 8.79% from FVC_MS_, close to 9.54% from the FVC_GP_ trend.

### 4.3. Spatial Distribution of Decreased Grasslands and the Degradation Hot Plots

**Figure 5:**
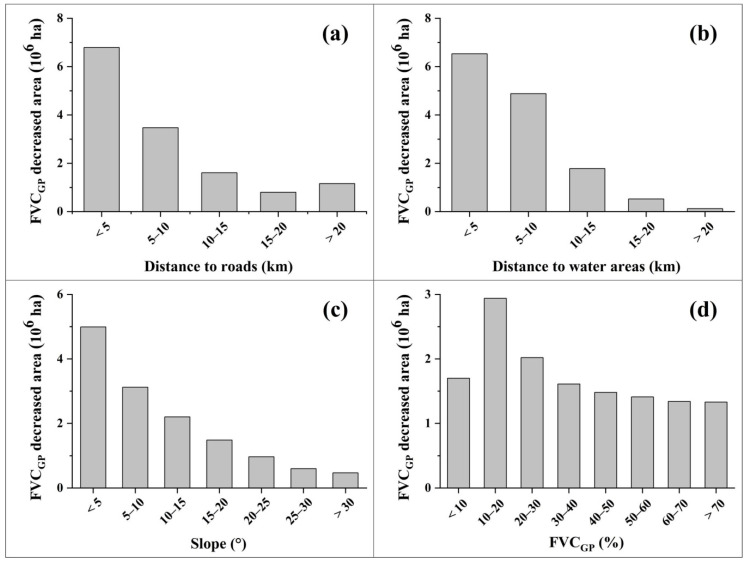
Spatial variations of FVC_GP_ decreased grassland areas in the TP with (**a**) distance to roads, (**b**) distance to water areas, (**c**) slope, and (**d**) FVC_GP_.

The buffering analyses found that the DE grassland type mostly occurred in the areas along roads and rivers, and surrounding lakes. Figure 5a,b indicate that the DE grassland area apparently decreased with increasing distance to roads and water areas (i.e., lakes and rivers). Statistic results showed that 74.19% (14.53%) of the DE grassland plots were distributed in the zones within 10 km (1 km) from roads (Figure 5a), and 82.55% (11.64%) were within 10 km (1 km) from water areas (Figure 5b). Of the total DE grasslands, 58.64% had a gentle slope below 10° (Figure 5c) and 48.17% had a low FVC_GP_ below 30% (Figure 5d).

**Figure 6:**
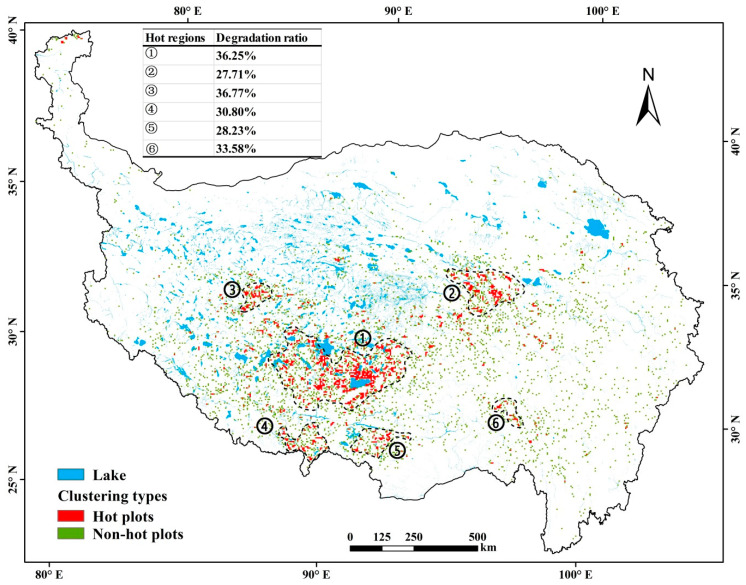
Spatial distribution of hot plots of grassland degradation in the TP during 2000–2019.

The clustering analysis results further revealed that the DE grassland was mostly in a point distribution pattern without significantly clustering characteristic (Figure 6). Statistics indicate that 72.97% of the DE grassland plots had a low or no clustering characteristic and were scattered in the IN grasslands. In 27.03%, totaling 3.74 Mha, the DE grasslands were significantly clustered, and densely distributed in 6 regions in south-center Tibet, south slope areas of the Himalayan Mountains, and the source areas of the Yangtze River and the Lantsang River (Figure 6). These 6 regions covered an area varying from 0.71 Mha, up to 10.46 Mha surrounding the Nam Co, comprising 27–37% of the DE grasslands, showing high risks of grassland degradation.

### 4.4. Influences of Climate and Grazing Intensity on Grassland Changes

**Figure 7:**
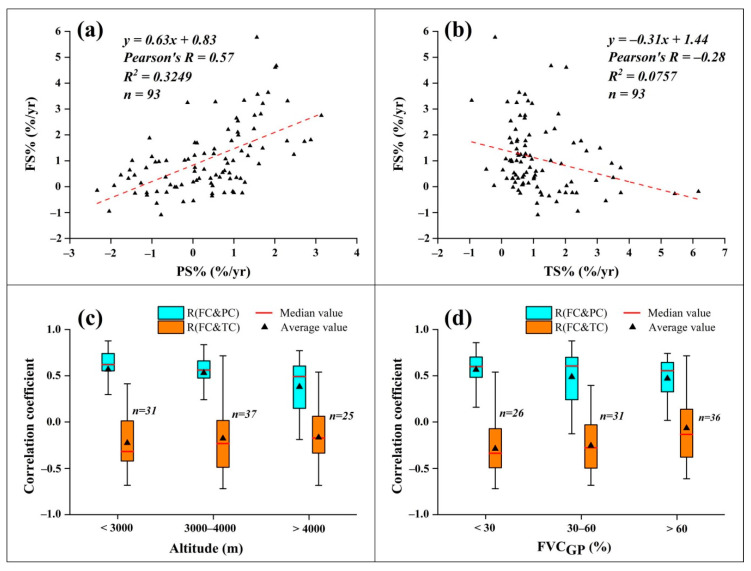
Correlation of grassland FVC_GP_ change rates (FS%) related to (**a**) precipitation change rate (PS%) and (**b**) temperature change rate (TS%) in the TP, and variation of the correlation coefficient of FVC_GP_ changes (FC) related to precipitation change (PC) and temperature change (TC), with (**c**) altitude and (**d**) FVC_GP_ gradients.

Correlation analyses indicate that the annual FVC_GP_ change was positively correlated with precipitation changes, while insignificantly and negatively correlated with temperature changes, with the correlation coefficient (R) of 0.57 (R^2^ = 0.3249) and −0.28 (R^2^ = 0.0757), respectively (Figure 7a,b). With increasing altitude, the R between precipitation and FVC_GP_ decreased, while the R between temperature and FVC_GP_ increased (Figure 7c). Using FVC_GP_ as an approximation of wet–dry conditions, we analyzed the R between FVC_GP_ and precipitation in different wet–dry gradients (Figure 7d) and found that it was higher in drier areas than wetter areas.

**Figure 8:**
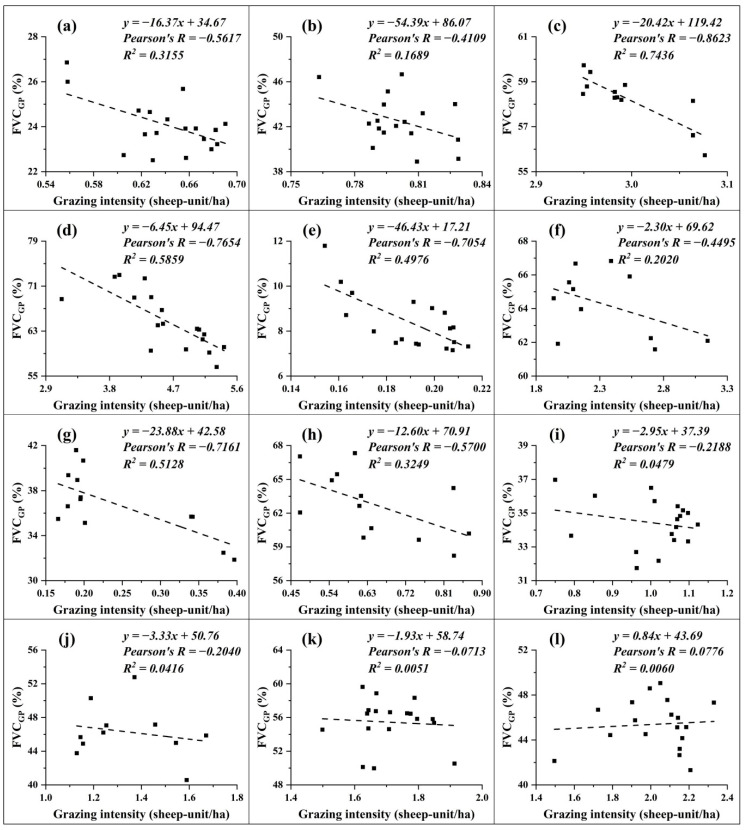
Correlation results between FVC_GP_ and grazing intensity at provincial (**a**,**b**) and county levels (**c**–**l**): (**a**) Tibet, (**b**) Qinghai, (**c**) Menyuan, (**d**) Zhouqu, (**e**) Rutog, (**f**) Dege, (**g**) Qumarlêb, (**h**) Chindu, (**i**) Basu, (**j**) Kangding, (**k**) Seni, and (**l**) Damxung.

Figure 8 presents annual variation of mean FVC_GP_ and grazing intensity in the TP during 2000–2018. The correlation analyses indicate that the annual grassland FVC_GP_ had a negative relationship with the mean annual grazing intensity at both provincial and county levels (Figure 8). At the provincial level, the correlation coefficients (R) in Tibet and Qinghai were −0.56 (R^2^ = 0.3155) and −0.41 (R^2^ = 0.1689), respectively (Figure 8a,b). At county level, similar correlation was found (Figure 8c–l). However, in the counties (Figure 8i–l) with precipitation decreasing and temperature rising, the grassland FVC_GP_ showed a slightly decreased trend, and the correlation was significantly lower.

From field investigations and herdsmen interviews, it was found that in the areas, especially in the northwest TP with less population and more grassland per capita, the livestock number has been evidently reduced due to the grassland protection policies well implemented, and the grass growth status is much better. However, in some densely populated areas with the policies not fully putting into effect yet, overgrazing and grassland degradation are still rather serious. The hot spots of grassland degradation are mostly distributed in such areas. The herdsmen reflected that the financial subsidy for grassland protection that is supplied according to grassland area at the same quota cannot compensate the income loss caused by the reduction of livestock number.

## 5. Discussion

### 5.1. Uncertainties in the Identification of Grassland Change Trends

Using RS-derived FVC time series data to identify grassland change trends and degrading remain uncertainties due to the differences in data sources, resolution, time span, and the shifts or degradation of RS sensors. The study results revealed that 62.12% of the grasslands in the TP increased and 9.54% decreased in FVC_GP_ during 2000–2019. Compared to the results of several other studies [8,14,34,35], the general trends of grassland dynamics are similar, but the proportion and extent of trend types are different. For example, our result of the FVC decreased grassland area is smaller than that of previous studies, e.g., 29.63% derived from MOD13A2 C6 (2000–2015, 1 km resolution) [8], 10.31% from SPOT-VEG (2000–2012, 1 km) [34], 38.27% from MOD13A2 C5 (2000–2015, 1 km) [8], and 51.63% from GIMMS-NDVI3g (2000–2012, 8 km) [34]. Following causes could be attributed to the large differences. The one is the difference of time span. The analyses in the above studies were done for the period up to 2015, and thus not including changes in the most recent years. From our analyses, the grassland FVC was obviously decreased from 2012 to 2015, and then largely increased since 2015 (Figure 3), probably because the forage–livestock balance policy was fully implemented in around 2014 and reduced the number of grazing animals. The second is the difference in data resolution. Previous studies indicate that the FVC trends from low-resolution GIMMS-NDVI3g cause more uncertainties than SOPT-VEG and MODIS data [34,38], particularly in mountain areas such as the TP [8]. The third is the sensor shifts or degradation. Some studies indicate that due to such causes, use of GIMMS-NDVI3g or MODIS C5 products caused underestimation of grassland area of FVC increase in the TP [8,34,36]. The sensor shifts of GIMMS-NDVI3g and SOPT-VEG data and the sensor decline of MODIS C5 data have compromised their ability to identify grassland dynamics [36,37,38]. The MODIS C6 data used in this study is deemed as a more reliable product at present [20,36,37,38,39,40].

The above comparisons imply that using imagery-based FVCs to assess grassland dynamics involves uncertainties. Even using same imagery, the results showed some differences when the period of FVC estimation is different (Table 4). Thus, the results should be interpreted with cautions and should be better checked by field observations. In addition, the identification of grassland trends should be better based on high-resolution imagery data, taking into account the spatial variations of grass growth season to estimate the grass coverage.

The grassland degrading areas in this study were simply identified based on the decline trend of FVC_GP_, not considering other aspects such as deterioration of soil properties [6,8], losses of dominant species [9,12], and invasion of nonedible weeds [32,33]. Thus, the degrading area of grasslands identified in this study might be partly underestimated. Additionally, the identified trends just reflected the process of grass coverage changes during the whole study period from 2000 to 2019, not differentiating the changes in different periods. By analyzing the annual variation of grass coverage, we found that in some DE grasslands, the FVC_GP_ showed an increasing trend in recent five years due to reduced grazing. These variations were not considered in this study, because huge computation is needed.

### 5.2. Heterogenety of Grassland Change Causes

Two major factors contributed to the grassland changes. One was change in climatic factors, particularly precipitation. In recent two decades, our analyses indicated that the precipitation increased in the north and decreased in the south TP, while most regions showed a warming trend, in consistence with the conclusion documented by previous studies [8,14,17]. Our analyses found that the influence of precipitation on the TP grassland changes was more significant than temperature, and the climatic influences on FVC_GP_ were spatially heterogeneous. Previous studies also found the similar effects of climate change on grassland coverage with altitude gradients [28,68]. This implies that the cooling caused by increased altitude had weakened the positive effects of precipitation on grassland growth, as in the high-altitude areas, the grassland growth is more restricted by low temperature [8,69]. Although this difference existed, precipitation could be a major factor driving the FVC_GP_ changes, as water availability is the main factor restricting grassland growth in the water-deficient areas [16,17]. With increasing humidity, the positive effects of precipitation and the negative impacts of temperature on grassland growth both decreased, in accordance with the results of previous studies [18,69].

With the implementation of grassland protection policies, the livestock number and grazing intensity have been decreased in the TP. The study results found that the grassland FVC_GP_ was negatively correlated with grazing intensity, implying that the grassland protection policy could be also an important contributor to the overall increase in the grassland coverage [15,20,70]. The county-level analyses showed that the correlation coefficients between FVC_GP_ and grazing intensity were relatively low in precipitation-decreasing and temperature-rising counties. This could be attributed to the negative impacts of decreased precipitation and increased temperature that may offset the contribution of reduced grazing intensity to some extent [8,15,19].

In addition, field investigation and herdsmen interview found that the grassland FVC showed an increase trend in the northwest TP with less population, while a decrease trend in more densely populated areas. This was due to the grassland protection policies in the former have been more effectively implemented than the latter, causing different grazing intensity and FVC trends. Moreover, field observations found that overgrazing surrounding the resident plots was rather common, which could be the reason why the grassland degradation showed a point-distribution characteristic in the TP.

### 5.3. Policy Implications

This study found that the grassland FVC in the TP was increased in most areas, partly benefited from the grassland protection policies that reduced the grazing intensity, suggesting that the policies are generally effective in promoting the grassland restoration. However, in some relatively densely populated areas, overgrazing and grassland degrading were still rather serious, because the financial support cannot compensate the income loss caused by the reduction of livestock number. This implies that the policies of financial support to reduce the grazing intensity may need some adjustments. Currently, the subsidy for achieving the balance between forage and livestock by reducing number of grazing animals, is supplied with the same quota of 22.5 CNY/ha according to grassland area, not considering the differences in grassland qualities and protection effectiveness. Therefore, it is suggested to increase the subsidy quota for high quality grasslands that are often distributed in relatively populated areas, and thus to improve the policy implementation effectiveness. In addition, more effective supervision and monitoring of grazing intensity could be important to achieve the aims of sustainable use of grassland and eco-security in the TP, particularly for the hot areas of grassland degradation.

Climate changes generally had an overall positive effect on the grassland restoration in the TP during the past decades, and would gain a further benefit, particularly in the cold areas [18,69], as the climate in the TP is highly possible to become warming and wetting in next few decades [8,42]. However, in the arid areas, continued warming may increase the frequency and intensity of droughts, and cause grassland degradation [16,68]. To mitigate the possible impact of climate changes, the government should enhance the financial support and monitoring to further reduce the grazing intensity in the areas the grassland still under degrading and in the arid areas.

## 6. Conclusions

In this study, the hot plots of grassland degradation in the TP were identified by quantifying trends of the annual average FVC in the green grass period (FVC_GP_), and the causal factors of grassland changes were detected to support regional grassland management. The results indicate that the majority of the grassland in the TP was increased or not changed in the FVC_GP_ during 2000–2019. Only 9.54% of the grassland was significantly decreased in the FVC_GP_ (−0.83%/year), mainly occurred in the areas along roads and rivers and surrounding lakes. Of the total FVC_GP_ decreased grassland, 73% was distributed in a point pattern, and 27% was significantly clustered and identified as hot plots of grassland degradation, mainly distributed in six main regions. Decreased grazing intensity and increased precipitation were the main contributors for the overall increase of grassland coverage in the TP, while local overgrazing could be the main cause for the coverage decrease. To alleviate the grassland degradation, the government supports and supervision should be strengthened to further mitigate the pressure of animal grazing, particularly in the hot plots of land degradation.

## Figures and Tables

**Table 1:** Classes and the criteria of grassland change trends.

Grassland Change Trends	Classification Criterion
Increased	Z > 1.96 or (0.675 ≤ Z ≤ 1.96 and FS% > 0.25)
Not changed	|Z| ≤ 0.675 or (0.675 ≤ |Z| ≤ 1.96 and |FS%| ≤ 0.25)
Decreased	Z < −1.96 or (−1.96 ≤ Z ≤ −0.675 and FS% < −0.25)

**Table 2:** Area, proportion, and mean Fractional Vegetation Cover in the green grass period (FVC_GP_) of grasslands with different green grass period (GP) in the TP during 2000–2019.

Green Grass Period (Month)	Area (Mha)	Proportion (%)	Mean FVC_GP_
<3	20.09	13.86	10.87
3–4	45.06	31.10	22.53
4–5	41.68	28.76	40.96
5–6	16.64	11.48	52.39
>6	21.46	14.80	53.25

**Table 3:** Area, proportion, FVC_GP_ and FS% of different grassland change trends in the Tibetan Plateau (TP) during 2000–2019.

Grassland Change Trends	Area (Mha)	Proportion (%)	FVC_GP_ (%)	FS% (%/year)
Increased	90.03	62.12	31.31	1.24
Not changed	41.07	28.34	40.02	0.08
Decreased	13.83	9.54	35.62	−0.83

**Table 4:** Comparison of composition (%) of FVC_MS_ and FVC_GP_ trends based on MOD13Q1 C6 data in the TP during 2000–2019.

Trend Types	FVC_MS_ Trends	FVC_GP_ Trends	Consistent Trends
Increased	63.62	62.12	57.71
Not changed	27.59	28.34	21.29
Decreased	8.79	9.54	7.36

## Data Availability

The data presented in this study are available on request from the corresponding author.
